# Insight into Greenhouse Gases Emissions and Energy Consumption of Different Full-Scale Wastewater Treatment Plants via ECAM Tool

**DOI:** 10.3390/ijerph192013387

**Published:** 2022-10-17

**Authors:** Yuhe Tian, Shuang Liu, Zheng Guo, Nan Wu, Jiaqi Liang, Ruihua Zhao, Linlin Hao, Ming Zeng

**Affiliations:** 1 College of Marine and Environmental Sciences, Tianjin University of Science & Technology, Tianjin 300457, China; 2 Key Laboratory of Green Process and Engineering, Institute of Process Engineering, Chinese Academy of Sciences, Beijing 100190, China; 3 School of Environmental Science and Engineering, Shandong University, Qingdao 266237, China; 4 College of Engineering and Technology, Tianjin Agricultural University, Tianjin 300384, China

**Keywords:** greenhouse gas emission, wastewater treatment, carbon, energy recovery, ECAM

## Abstract

Greenhouse gas (GHG) production is one of the urgent problems to be solved in the wastewater treatment industry in the context of “carbon neutrality”. In this study, the carbon emissions and energy consumption of typical wastewater treatment processes in China were evaluated, starting from different cities and water treatment plants. Tool of Energy Performance and Carbon Emission Assessment and Monitoring (ECAM) was used. By comparing the influent BOD_5_, it was found that the energy consumption for wastewater treatment was positively correlated with the influent organic load. The annual CH_4_ emission of Xi’an WWTP can reach 19,215 t CO_2_eq. Moreover, GHGs are closely related to the wastewater treatment process chosen. WWTP B of Kunming used only an anaerobic process without continuous aeration, with an average monthly energy consumption of 8.63 × 10^5^ kW·h. The proportion of recoverable biogas was about 90% in the GHG discharged by the traditional process. However, the anaerobic digestion-thermoelectric cogeneration process can make the recovery of the biogas utilization ratio reach 100%. Compared to the Shuozhou WWTP and WWTP A of Kunming, the Strass WWTP served the smallest population and had the largest treatment capacity, reaching the lowest energy consumption, consuming only 23,670 kW·h per month. The evaluation and analysis of ECAM provide data support and research foundation for the wastewater treatment plants to improve energy utilization and reduce greenhouse gas emissions.

## 1. Introduction

Sewage is the carrier of resources and energy, which contains a large amount of organic matter [1]. Organic matter is an energetic substance, and sewage also contains a large number of plant nutrients, which contains great chemical energy and thermal energy. However, traditional wastewater treatment usually separates, degrades and converts pollutants in water through various complex artificial technical means, regardless of the consumption of resources and energy, which is a process of “energy consumption” and “pollution transfer” [2,3]. At the same time, biosorption is being developed as an alternative treatment method to replace traditional remediation methods [4,5]. According to statistics, Chinese carbon emissions ranked the forefront of the world. In 2014, Chinese urban sewage production was 5.546 million m^3^, wastewater treatment energy consumption accounted for 0.73% of the total electricity consumption, and greenhouse gas (GHG) emissions reached 91 million tons. Cities are the most important units for carbon and local air pollutants emission accounting, statistics and management [6]. Studies have shown that wastewater treatment volumes are as high as 200 million cubic meters per day and that carbon emissions from wastewater treatment systems account for 1% to 2% of total carbon emissions, more than 25% of global carbon emissions, with a growing trend [7,8,9,10]. The municipal wastewater treatment industry is a major energy source in China, occupying a large amount of energy and producing a large number of greenhouse gases. Under the current situation of double improvement of sewage collection and pollutant removal ratio, the energy consumption problem of wastewater treatment plant (WWTP) is becoming more and more prominent and serious [11].

Under the background of carbon neutralization, the carbon neutralization operation of low-carbon urban domestic sewage plants is undoubtedly the future development direction of the wastewater treatment industry. The total amount of carbon dioxide directly or indirectly emitted by WWTPs in a certain period of time is offset by self-produced clean energy, adopting low-energy treatment process, and implementing energy-saving transformation of equipment, so as to realize the net “zero emission” of GHGs in WWTPs, which has the advantages of low energy consumption, low emission, and sustainability [12,13]. The latest research progress of carbon neutralization technology in WWTPs was investigated, and the energy consumption value was analyzed, in order to provide reference for the realization of carbon neutralization operation of urban WWTPs in China.

Some studies show that the influent COD concentration of municipal wastewater is about 250–950 mg/L. Depending on the COD load consumed, different calculations have different results. But in general, its potential energy value is 1.66–1.93 kW·h/m^3^ (COD = 430–500 mg/L) or as high as 3.09–3.86 kW·h/m^3^ (COD = 800–1000 mg/L) [14]. The typical energy consumption of activated sludge process (CAS) containing nitrification process is 0.35–0.80 kW·h/m^3^, which has broad research prospects for the recovery of residual energy in the realization of low carbon WWTP [15].

The so-called low-carbon wastewater treatment is the realization of carbon neutralization by wastewater treatment. It refers to the total amount of carbon dioxide directly or indirectly emitted by WWTPs in a certain period of time, which is completely offset by the production of clean energy, the use of low-energy treatment technology, and the implementation of the energy-saving transformation of equipment, so as to realize the net “zero emission” of GHGs in WWTPs. In recent years, the research shows that the practical ways of carbon neutralization in WWTPs mainly include the utilization of sewage temperature heat energy, sludge cogeneration, carbon source capture of raw water, solar photovoltaic power generation, application of low-energy wastewater treatment process, and energy saving transformation of high-energy equipment [16,17]. For the vision of future water recycling plants, Suez company analyzed its 30 sewage plants operating in France and summarized five directions: energy efficiency optimization, nutrient recovery, sludge reduction and reuse, bio-gas recycling, and production of new substances [18].

Exploring the sustainability of WWTPs in terms of energy recyclability and environmental impact is now the novel challenge. By evaluating the service population and treatment efficiency of different WWTPs, it is possible to compare their performance and determine the optimal operation, thereby reducing carbon emissions and achieving carbon neutrality.

The purpose of this study is to: (1) Explore the impact of city size on carbon emissions and energy consumption; (2) evaluate the energy consumption and GHG emission of different biological treatment processes based on modified A^2^/O oxidation ditch process; (3) compare the domestic plants in China with foreign treatment plants that combine anaerobic digestion.

## 2. Materials and Methods

### 2.1. Study Area

**Figure 1:**
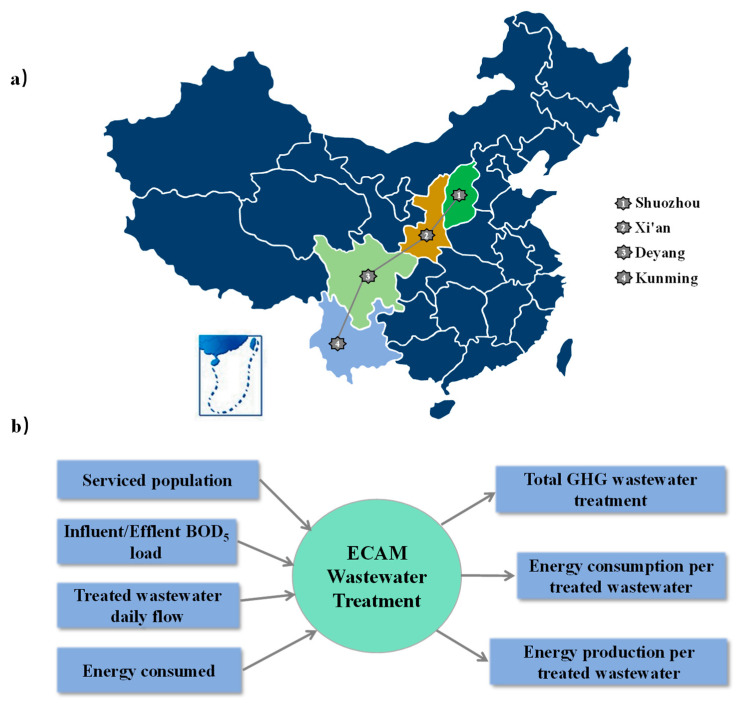
Urban location of wastewater treatment process (**a**) and the main input and output data of ECAM tool (**b**).

In this study, four cities of Shuozhou, Xi’an, Deyang and Kunming from north to south China were selected. Figure 1a shows the geographical relationship of cities in four provinces. Because the terrain and location of the selected cities were different, they can be compared. When comparing and analyzing the energy consumption and carbon emissions of wastewater treatment processes in Shuozhou, Xi’an and Deyang, it was possible to evaluate which scale and region of the city were more suitable for a particular wastewater treatment process.

Shuozhou is located in the north of Shanxi Province, surrounded by mountains on three sides with high terrain, and the total area of 106,000 km^2^ and resident population of 1,593,444. Xi’an is located in the core position of Shaanxi Province, with a total area of 10,800 km^2^ and a permanent population of 13,163,000. Because there are great differences in topography between east and west and complex terrain, Deyang is chosen which locates in the plain with a total area of 60,000 square kilometers and a permanent population of 3,456,161. Kunming is the city with the lowest mid-latitude in the selected city, with a total area of 21,000 km^2^ and a permanent population of 8,460,088.

### 2.2. The ECAM Tool

The Energy Performance and Carbon Emission Assessment and Monitoring (ECAM) tool was used to evaluate the GHG emissions in different stages of wastewater treatment. Through several field visits and investigations, researchers recorded the operation of electrical equipment in WWTPs and the data of energy estimation under different actual operation conditions, analyzed different stages of the wastewater treatment system [19]. According to the retrieval of ISI knowledge network, the construction data of ECAM model came from five published pieces of literature [19,20,21,22,23].

The ECAM tool allows water utilities and users to assess performance in terms of greenhouse gas emissions and relative weight at various stages and to identify potential areas for improvement, particularly in terms of energy savings. The summary of the input and output data of ECAM is shown in Figure 1b. According to the Intergovernmental Panel on Climate Change (IPCC) and International Water Association (IWA) system evaluation, by inputting the original data such as population, water quantity, energy estimation and wastewater treatment type, the GHG and energy estimation values of the water treatment process were calculated [24].

The assessment tool can help the water department to conduct self-help preliminary analysis of potential project investment. The ECAM tool set the baseline for energy estimates, identified key areas for reducing GHG emissions, highlighted cost-saving points and provided a global carbon reduction map for water agencies [25]. It can modify some basic parameters by itself, and make the benchmark analysis of greenhouse gas emissions according to the actual situation of its own region or project. However, this tool also had many limitations. For example, the detailed calculation of the energy consumption and carbon emissions of a particular structure of the water treatment process is yet to be resolved, and the carbon emissions of the sludge treatment process are one of the technologies that need to be improved in the future [26].

## 3. Results and Discussions

In this study, three schemes were compared to evaluate the energy estimation and carbon emissions of wastewater treatment process in different cities and processes, to explore their law of energy estimation and carbon emissions. The comparison results of the three schemes were listed in Table 1.

### 3.1. Scheme 1: Impact of City Scale on Carbon Emissions and Energy Consumption—Energy Estimation and GHG Emission in Different Cities

The service population of WWTPs was positively correlated with the urban population, which helped to analyze the differences in energy consumption and GHG emissions when the same process was applied to different cities. Scheme 1 focused on the traditional process and widely used A^2^/O process. In order to make the analysis representative, the domestic cities were divided into three levels according to the population. The population of the first-level city was more than 500,000 people, taking an example of Xi’an WWTP serving population of 500,000. The second-level city was 300,000–500,000 people, taking the example of Deyang WWTP serving population of 340,000. The third-level city was 100,000–300,000 people, taking an example of Shuozhou WWTP serving population of 200,000. The WWTP of these cities owns the traditional A^2^/O process.

#### 3.1.1. Data Input of ECAM

The detailed data of water inflow, water quality, and energy consumption of the three municipal WWTPs are shown in Table 2. Through the input of the original data, the power consumption of WWTPs in Xi’an, Deyang, and Shuozhou was 4.94 × 10^5^ kW·h, 5.02 × 10^6^ kW·h, and 3.35 × 10^6^ kW·h, respectively. Under the same process conditions, the influent quality of each WWTP was different. The influent BOD_5_ of Xi’an WWTP with the largest service population can reach 35,200 kg per year, while the influent BOD_5_ of Shuozhou WWTP with the smallest service population was 1800 kg per year. It can be seen that the energy consumption of wastewater treatment was positively correlated with the influent organic load. When sufficient organic matter was provided, the water treatment reaction can be fully carried out without providing additional energy. Thus, if the WWTP was to achieve the goal of energy conservation and emission reduction, it was necessary to ensure sufficient organic supply in the influent to reduce energy consumption.

#### 3.1.2. Outputs of the Calculated GHG by ECAM

**Figure 2:**
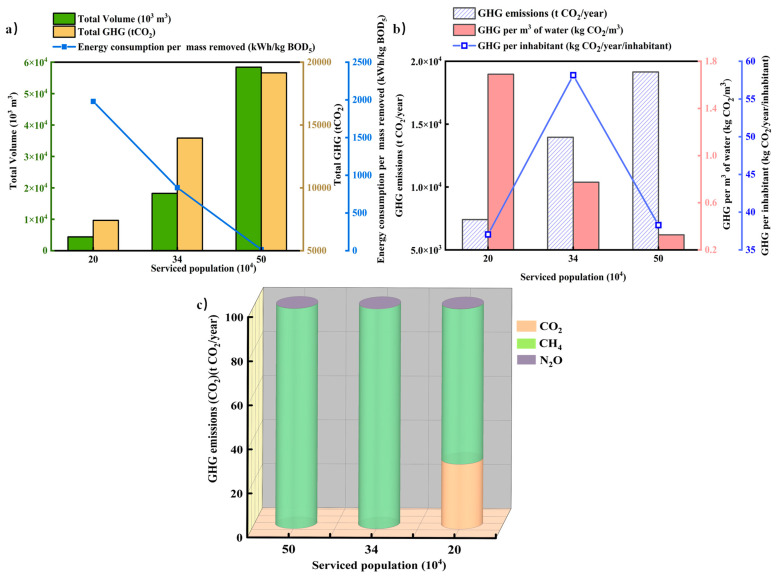
Relationship between population and water, energy consumption and GHG emissions in municipal wastewater treatment processes (**a**), the relationship between population and per capita GHG in municipal wastewater treatment process (**b**), and proportion of GHG emitted from municipal wastewater treatment processes (**c**).

The ECAM tool evaluated the carbon emissions based on the detailed information of each WWTP. Figure 2a shows that the amount of wastewater treatment increased with the growth of the population. Xi’an the first-level city with the largest permanent population had the largest annual treatment capacity, reaching 58.4 million m^3^. In addition, the second-level city of Deyang and the third-level city of Shuozhou had annual treatment capacities of 18.02 and 4.38 million m^3^, respectively.

The relationship between population and per capita GHG in the municipal wastewater treatment process was demonstrated in Figure 2b. According to the analysis of carbon dioxide (CO_2_) emission per person per year in the evaluation data, the annual GHG emission per capita in the service area of a WWTP in Deyang was the highest in three cities and the annual GHG emission per person was estimated to be 58.16 kg CO_2_. However, Xi’an WWTP and Shuozhou WWTP the annual GHG emissions per person were estimated to be 38.29 kg CO_2_ and 37.05 kg CO_2_, respectively. The comparison displayed the annual inflow BOD_5_ of 6500 kg in Deyang WWTP, which was higher than that of Shuozhou with BOD_5_ of 1800 kg when the treatment process and service population were similar. It can be concluded that the emission of greenhouse gases was related to the influent quality. The higher the organic load in the influent quality, the higher the carbon emissions were likely to be.

In the process of wastewater treatment, the GHGs which lead to the increase in carbon emissions were mainly composed of methane (CH_4_), CO_2_, and nitrous oxide (N_2_O). From the GHG composition analysis of three urban WWTPs in Figure 2c, CH_4_ was the dominant one, and the annual CH_4_ emission of a WWTP in Xi’an can reach 19,215 t CO_2_eq. The N_2_O emissions from WWTPs in three levels of cities were the least GHGs can reach 777 t CO_2_eq. Shuozhou WWTP had the least service population and the smallest influent BOD_5_ load. The proportion of CH_4_ emission was relatively small, while the proportion of CO_2_ emission increased, the value of GHG emissions was 325 t CO_2_/year. As a new energy, CH_4_ had great recycling value, which had a potential role in promoting the realization of energy conservation and emission reduction targets. It can be seen that a large number of CH_4_ emitted can be fully recovered. How to realize the recovery of CH_4_ and control various types of greenhouse gases was closely related to the water treatment process used.

The amount of GHGs emitted by the three WWTPs in each stage of the wastewater treatment process has been evaluated in Table 3. Wastewater treatment process, sludge treatment process, sewage discharge process, and power consumption process will emit a certain amount of GHGs. The evaluation results show that the amount of greenhouse gases emitted by the wastewater treatment process was the largest. This was because the selected A^2^/O process in the reaction process was anaerobic, anoxic, aerobic alternative operation.

### 3.2. Scheme 2: Feasibility Evaluation of Different Biological Treatment Processes—Energy Consumption Estimation and GHG Emission of Modified A^2^/O Oxidation Ditch Process

Although the A^2^/O oxidation ditch process can achieve high standard effluent, it will consume a lot of energy. Therefore, in order to take full advantage of this process, it was necessary to estimate and analyze the emissions. Full consideration needed to be given to the recovery and use of energy and to assessing the value of recovered energy. Under the condition of controlling the relevant factors analyzed in this study, it was necessary to improve the existing process to further save energy and reduce carbon emissions. Hence on the basis of traditional A^2^/O process, Scheme 2 of this study analyzed the energy estimation and GHG emission of the improved A^2^/O oxidation ditch process.

#### 3.2.1. Improving the Selection of A^2^/O Processes

So as to judge whether the improved process had outstanding advantages, WWTP with different processes in a certain area of Kunming was selected. The independent variable of controlling the quality of inlet and outlet water was the same, and different processes were changed. At the same time, the GHG emission coefficient also changed. The energy estimation difference and GHG emission of improved A^2^/O oxidation ditch, anaerobic, and aerobic processes were analyzed. ECAM online assessment tool was used to evaluate the composition of GHG emissions and to analyze the proportion of recyclable gases, which afforded a method to determine whether a process had the value of recycling energy. If it was confirmed that the new energy gases such as biogas produced by this process had reached a certain level and had the value of energy recovery, the biogas produced by anaerobic digestion, gas collection and cogeneration can be recovered. In the era of advocating carbon neutralization, it was undoubtedly the preferred way to get twice the result.

**Figure 3:**
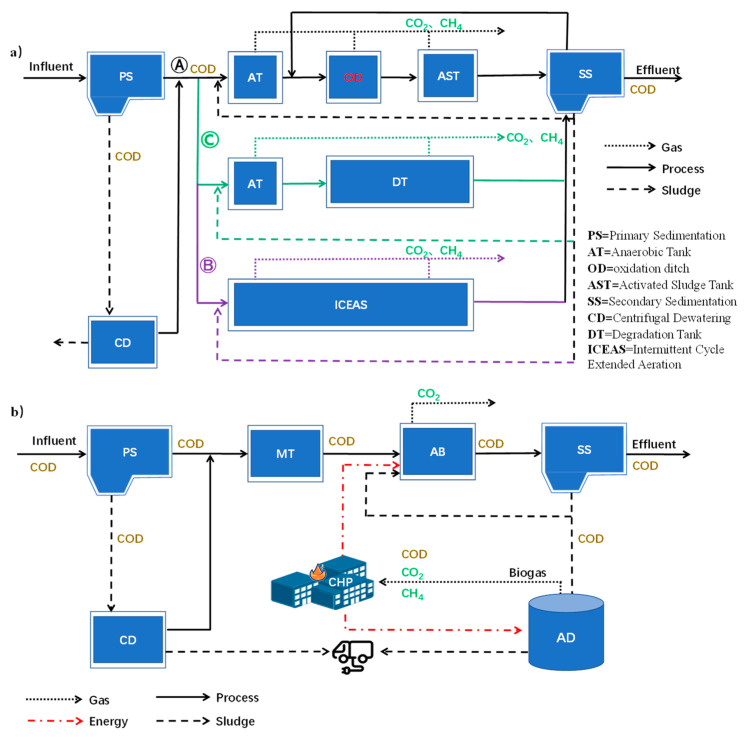
Energy flow and gas emission diagram of improved A^2^/O oxidation ditch process, process flow (**a**): Ⓐ WWTP A of Kunming, Ⓑ WWTP B of Kunming and Ⓒ WWTP C of Kunming, process flow, energy flow and gas emission diagram of Strass wastewater treatment plant in Austria (**b**).

As shown in Figure 3a, the direct carbon emission process in the A^2^/O process was a biodegradation process. Studies had shown that CH_4_ usually had the highest yield in the anaerobic stage, which was because the anaerobic tank provided a strict anaerobic growth environment for methanogenic bacteria, and the degradation of organic matter releases CH_4_ and CO_2_ into the environment [27]. Under anoxic conditions, ammonia nitrogen in water was oxidized to nitrite and nitrate, and CO_2_ was released. N_2_O was produced by nitrification in the anaerobic tank and anoxic tank dissolved in water and released under aeration agitation in the aerobic zone. N_2_O production increased when the oxygen supply was insufficient [28]. N_2_O production of three WWTPs was the least in GHG, which showed that the aeration is sufficient. Most countries consider anaerobic as a less costly and efficient way to recycle CH_4_ [29]. A WWTP in Kunming gave this idea, the oxidation ditch process into A^2^/O adopted the improved A^2^/O oxidation ditch process.

For the purpose of analyzing the outstanding characteristics of the improved A^2^/O oxidation ditch process, due to the same service area, it was assumed that the inlet and outlet water quality of each WWTP was the same, the process flow was changed, and the energy consumption and GHG emission of various processes were compared. The improved A^2^/O oxidation ditch process was used in WWTP A as shown in Figure 3a(Ⓐ). WWTP B adopted an anaerobic process as shown in Figure 3a(Ⓑ) and WWTP C used an activated sludge intermittent cycle extended aeration (ICEAS) process as shown in Figure 3a(Ⓒ). The improved A^2^/O oxidation ditch process provided longer hydraulic retention time, improved ammonia nitrogen oxidation efficiency and higher reductase activity of ammonia-oxidizing bacteria and nitrite-oxidizing bacteria in activated sludge [30]. Ammunition, nitrification, and denitrification reactions were more thorough, which can fully convert carbon in organic matter into CH_4_ for further recycling.

#### 3.2.2. Data Input of ECAM

**Figure 4:**
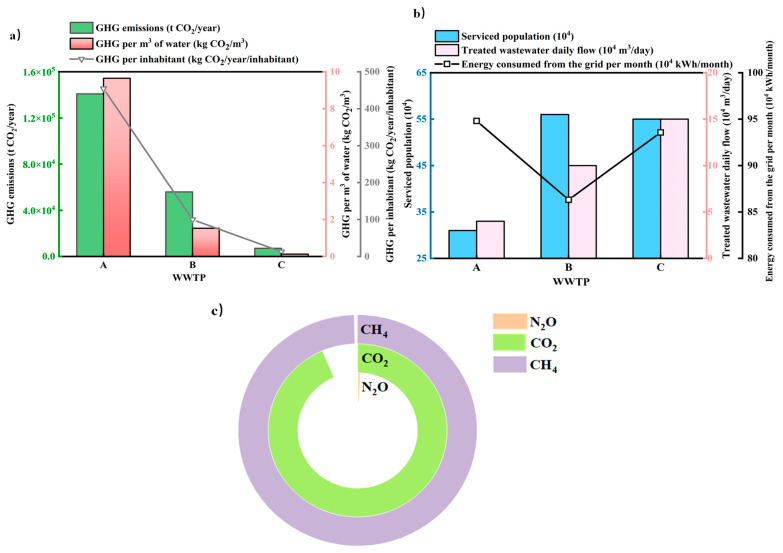
Relationship between service population, treated water quantity, and energy consumption in three Kunming WWTPs (**a**), relationship between GHG emission and treatment water in three Kunming WWTPs (**b**), and GHG emission composition and proportion of A WWTP (**c**).

Table 4 were the basic data of three WWTPs that input ECAM. Among the three WWTPs, WWTP A had the least serviced population, only 310,000 people. In addition, WWTP B and WWTP C had similar service populations, 550,000 and 560,000 respectively. There was a roughly positive correlation between the wastewater treatment capacity of the three plants and the service population. From Figure 4a, it can be clearly seen that the sewage volume will also increase with the increase in the service population.

By analyzing the energy consumption WWTP A, B, and C of Kunming, it was found that the energy consumption of plant B was significantly lower than that of plants A and C. The average monthly energy consumption of plant B was 8.63 × 10^5^ kW·h, while the average monthly energy consumption of plant A and C was 948,101.583 kW·h and 9.36 × 10^5^ kW·h, respectively. This was due to the fact that plants A and C require a large amount of aeration to maintain the aerobic environment, while plant B only adopted anaerobic process without continuous aeration, which reduced energy consumption. However, the lack of aerobic environment was easy to lead to incomplete nitrification and incomplete decomposition of organic matter. As a consequence, the anaerobic process alone was suitable for small WWTPs with low effluent quality requirements. WWTP A served significantly more people than WWTP B and WWTP C; however, energy consumed from the grid per month was the highest as seen in Table 4.

#### 3.2.3. Outputs of the Calculated GHG by ECAM and Energy Recycling Analysis

The analysis in Section 3.2.1 shows that GHG emissions were positively correlated with population in different levels of cities, but GHG emissions were obviously not correlated with the population for different WWTPs in the same city. The service population of Kunming Plant A was the smallest, but it can be seen from Figure 4a that the carbon emission of Plant A was the highest. Specific values were collated in Table 5. The GHG emission was 1.41 × 10^8^ kg CO_2_eq per year on average. The carbon emission of the water treatment process accounted for the majority, which was 1.52 × 10^8^ kg CO_2_eq. The GHG emission of energy consumption process accounted for the second, which was 1.13 × 10^7^ kg CO_2_eq. It was visible that in the same area, GHG emissions are closely correlated with the selected wastewater treatment process. Among them, the GHG emissions of aerobic process were the least, followed by the anaerobic process, and the GHG emissions of aerobic combined with anaerobic and anoxic mixed process were the most.

For WWTP A of Kunming with the largest GHG emission, the GHG composition was mainly composed of CH_4_, CO_2_, and N_2_O, which is shown in Figure 4c. The largest gas emission was CH_4_. In various processes of wastewater treatment, the CH_4_ emission from Kunming WWTP of A was 1.52 × 10^8^ kg CO_2_. CO_2_ emissions followed by 1.13 × 10^7^ kg CO_2_ and the least emission was N_2_O, only 6322 kg CO_2_. As a new energy gas, CH_4_ had the value of recycling. At present, the WWTPs generally begin to adopt the combined process of anaerobic digestion and cogeneration and use CH_4_ to generate heat energy or convert it into kinetic energy, which is provided to other process parts of the WWTP.

Qingdao WWTP used the process to convert the energy generated by biogas into heat sources and gas sources for sludge tanks and pumps. After the renovation of the WWTP in 2018, the water treatment process collected 6.11 × 10^6^ Nm^3^·a^−1^ CH_4_ [31], which is equivalent to 4.38 × 10^6^ t CH_4_. According to the IPCC Guidelines for National Greenhouse Gas Inventories, 1 t CH_4_ = 21 t CO_2_eq was used to calculate the CO_2_ equivalent of GHGs. The collected CH_4_ was equivalent to 2.08 × 10^8^ t CO_2_, and the power generation was 1.02 × 10^8^, providing more than 60% of the power consumption of the whole plant. The Kakolanmäki WWTP in Finland used the heat exchange of effluent and the energy generated by methane as the main energy sources of the WWTP. The annual energy consumption of the WWTP was 12,755 MW·h, and the energy recovered from biogas can reach 21,935 MW·h, achieving 100% recovery and utilization of energy [32].

### 3.3. Scheme 3: A Comparison of Domestic and International Low-Carbon Wastewater Treatment Processes—Energy Analysis and Carbon Emission Assessment of Low-Carbon Wastewater Treatment Processes Considering Energy Recovery

As there were few cases of recycling biogas and generating energy in China, scheme 3 selected the Strass WWTP in Austria, the pioneer of carbon neutralization, as the representative of biogas recycling. The plant was a WWTP where the dominant method was the “AB” method and the side flow process was low carbon, low consumption anaerobic ammonia oxidation process. It was an early pioneer in the water treatment industry in terms of carbon neutrality. The main process flow and energy flow were shown in Figure 3b. By comparing with the Shuozhou WWTP with the best energy utilization ratio in scheme 1 and the Kunming A wastewater treatment station with the improved process in scheme 2, the energy estimation direction was analyzed. The energy estimation value and GHG emission value evaluated by ECAM tool were used to evaluate the contribution value of recycling new energy gas to energy conservation and emission reduction and low carbon wastewater treatment process construction.

#### 3.3.1. Data Input of ECAM

**Figure 5:**
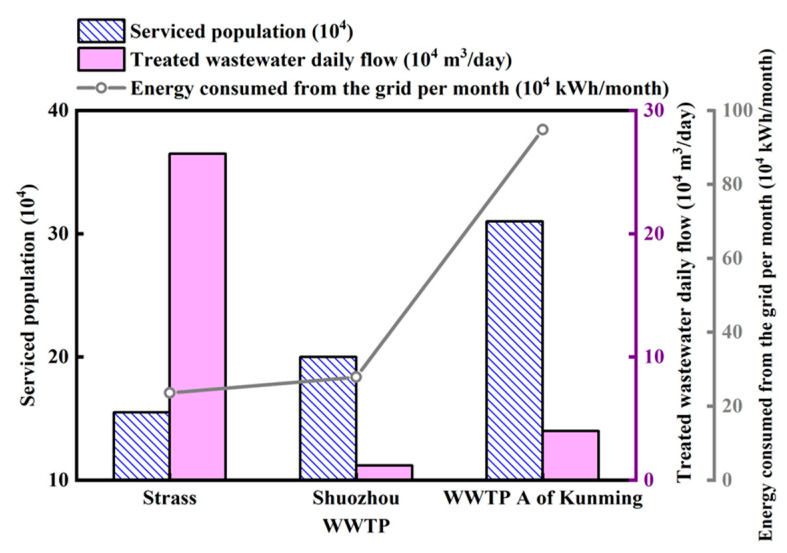
The relationship among service population, treatment capacity and energy consumption of different WWTPs in different regions.

For the sake of verifying the contribution of the new process in achieving low carbon and low energy consumption, this study used ECAM tool to evaluate the energy consumption and carbon emissions of Strass WWTP, Shuozhou WWTP, and WWTP A of Kunming. The initial data of the three WWTPs input ECAM tools were shown in Table 6. The permanent population of Strass WWTP fluctuated greatly, about 60,000 in summer and 250,000 in winter, serving a population of about 155,000. The previous analysis showed that the energy consumption of wastewater treatment was positively correlated with the service population and treatment capacity when the wastewater treatment process was the same. When the wastewater treatment process and the location of the WWTP were different, as shown in Figure 5, the population of the three WWTPs increased in turn. Although the service population of the Strass WWTP was the least, the treatment capacity was the largest, up to 26,500 m^3^/day. The treatment capacity of Shuozhou WWTP and A WWTP was only 12,000 m^3^/day and 40,000 m^3^/day, respectively.

#### 3.3.2. Outputs of the Calculated GHG by ECAM

Table 7 shows the assessment of GHG emissions at each stage of the wastewater treatment process of the three wastewater treatment plants. The output results showed that Shuozhou WWTP and A WWTP produced the most GHG during wastewater treatment, which was consistent with the conclusion in Section 3.1. However, Strass WWTP consumes the most electricity, reaching 8.59 × 10^7^ kg CO_2_eq/year. This is because the energy consumption of Strass WWTP was the least among the three WWTPs, which consumed only 236,070 kW·h per month. The core of achieving the goal of “energy saving” was that it used anaerobic digestion-thermoelectric cogeneration technology to recover CH_4_ produced in the water treatment process and convert it into energy reuse. In 2005, the total energy consumption of each unit in Strass WWTP was 2.87 × 10^6^ kW·h, the calorific value of CH_4_ was 35.9 MJ/m^3^, and the conversion efficiency of electric energy was 2.3 kW·h/m^3^. The capacity/energy consumption can reach 40% after replacing the cogeneration unit, and about 14% of the aeration can be saved [33,34].

## 4. Conclusions

In summary, the ECAM tool used in this study can be used in other cities in China, and even cities around the world. First, it was found that wastewater treatment energy consumption was positively correlated with the influent organic load. The ratio of treated wastewater daily flow to influent BOD was more than 4.5. Next, by comparing the conventional process with the novel low carbon process, the mixed process combining aerobic with anaerobic and anoxic emitted the most GHG, and the GHG emissions of aerobic process was the least. Finally, the energy production per treated wastewater of Stass WWTP was 0.32 kW·h/m^3^. The energy consumption of Xi’an WWTP and Deyang WWTP was 0.0085 kW·h/m^3^ and 0.28 kW·h/m^3^, respectively. If the energy recovery process was applied to the process improvement of the above two WWTPs, both can achieve 100% energy recovery with a view to achieving energy self-sufficiency. The collection of GHGs and the recycling of energy provide novel ideas and reference directions for the transformation of wastewater treatment plants to low carbon.

The limitation of this paper is that the accuracy and applicability of carbon emission assessment also depend on the accuracy of the methods used and the input data, because the carbon emission of sewage treatment plants depends largely on the operation of wastewater treatment processes. If the measured data are inaccurate, resulting in errors in the input data, the use of ECAM online assessment tools may lead to incorrect carbon emission assessment, thus deviating from reality.

## Figures and Tables

**Table 1:** Comparison results of the three schemes.

Scheme	Different	Results
Scheme 1	Impact of city scale	Carbon emissions were positively correlated with service population level
Scheme 2	Impacts of different biological treatment processes	The GHG emissions of aerobic combined with anaerobic and anoxic mixed process were the most, followed by the anaerobic process, and the GHG emissions of aerobic process were the least.
Scheme 3	Comparison of Chinese WWTP with international low-carbon WWTP	The studied national WWTPs produce more greenhouse gases than Strass WWTP

**Table 2:** The initial GHG evaluation stage is input into the ECAM tool.

Description	Xi’an	Deyang	Shuozhou	Unit
Resident population	500,000	340,000	200,000	People
Population connected to sewers	500,000	340,000	200,000	People
Serviced population	500,000	340,000	200,000	People
Treated wastewater daily flow	160,000	50,000	12,000	m^3^/day
Volume of treated wastewater	58,400,000	18,025,000	4,380,000	m^3^
Influent BOD_5_ load	35,200	6500	1800	kg
Effluent BOD_5_ load	1600	500	110.16	kg
Total Nitrogen load in the influent	6400	1250	510	kg
Total Nitrogen load in the effluent	2400	250	51.24	kg
CH_4_ emission factor (treatment)	0.12	0.12	0.12	kg CH_4_/kg BOD
N_2_O emission factor (treatment)	0.0045	0.0045	0.0045	kg N_2_O-N/kg N
CH_4_ emission factor (discharge)	0.021	0.021	0.021	kg CH_4_/kg BOD
N_2_O emission factor (discharge)	0.005	0.005	0.005	kg N_2_O-N/kg N
Energy consumed from the grid	494,674.56	5,018,750	3,346,617.6	kW·h
Emission factor for grid electricity	0.097	0.097	0.097	kg CO_2_eq/(kW·h)
Sludge removed from wastewater treatment	5,931,250	3,547,800	2,372,500	dry weight/kg
BOD_5_ removed as sludge	4,745,000	3,547,800	1,898,000	kg
Energy consumed from the grid per month	41,222.88	418,229.17	278,884.8	kW·h/month

**Table 3:** Detailed greenhouse gas assessment data of ECAM in wastewater treatment stage of Xi’an, Deyang and Shuozhou.

Description	Xi’an	Deyang	Shuozhou	Unit
Electricity (indirect)	47,983	486,819	324,622	kg CO_2_eq/year
Treatment process	−19,202,497.37	−14,445,869.89	−7,735,421.28	kg CO_2_eq/year
Discharged water	6762	942.4	198.6	kg CO_2_eq/year
Total GHG wastewater treatment	−19,147,752.11	−13,958,108.78	−7,410,600.75	kg CO_2_eq/year

**Table 4:** Digital data to the rapid assessment stage of the ECAM tool—WWTP in Kunming.

Description	WWTP A	WWTP B	WWTP C	Unit
Resident population	310,000	560,000	550,000	People
Population connected to sewers	310,000	560,000	560,000	People
Serviced population	310,000	560,000	560,000	People
Treated wastewater daily flow	40,000	100,000	150,000	m^3^/day
Volume of treated wastewater	14,600,000	36,500,000	54,750,000	m^3^
Influent BOD_5_ load	30,000	30,000	30,000	kg
Effluent BOD_5_ load	4500	4500	4500	kg
Total Nitrogen load in the influent	4500	4500	4500	kg
Total Nitrogen load in the effluent	2700	2700	2700	kg
CH_4_ emission factor (treatment)	0.12	0.48	0.018	kg CH_4_/kg BOD
N_2_O emission factor (treatment)	0	0	0.016	kg N_2_O-N/kg N
CH_4_ emission factor (discharge)	0.021	0.021	0.021	kg CH_4_/kg BOD
N_2_O emission factor (discharge)	0.005	0.005	0.005	kg N_2_O-N/kg N
Energy consumed from the grid	11,377,219	10,358,000	11,227,590	kW·h
Emission factor for grid electricity	0.997	0.997	0.997	kg CO_2_eq/kWh
Sludge removed from wastewater treatment	3,677,375	5,110,000	8,531,875	dry weight/kg
BOD_5_ removed as sludge	3,677,375	4,088,000	6,825,500	kg
Energy consumed from the grid per month	948,101.583	863,166.667	935,632.5	kW·h/month
Monthly energy costs	63,206.78	57,544.44	62,375.5	USD/month

**Table 5:** Detailed greenhouse gas assessment data of ECAM in wastewater treatment stage—WWTP A of Kunming.

Description	Current Value	Unit
Electricity(indirect)	11,343,087	kg CO_2_eq/year
Treatment process	−152,222,760	kg CO_2_eq/year
Sludge management (kgCO_2_eq/year)	0	kg CO_2_eq/year
Discharged water (kgCO_2_eq/year)	9535	kg CO_2_eq/year
Total GHG wastewater treatment	−140,870,137.80	kg CO_2_eq/year

**Table 6:** The initial GHG evaluation stage input into the ECAM tool.

Description	Strass	Shuozhou	WWTP A of Kunming	Unit
Resident population	60,000~250,000	200,000	310,000	People
Population connected to sewers	155,000	200,000	310,000	People
Serviced population	155,000	200,000	310,000	People
Treated wastewater daily flow	26,500	12,000	40,000	m^3^/day
Volume of treated wastewater	9,672,500	4,380,000	14,600,000	m^3^
Influent BOD_5_ load	7711.5	1800	30,000	kg
Effluent BOD_5_ load	397.5	110.16	4500	kg
Total Nitrogen load in the influent	689	510	4500	kg
Total Nitrogen load in the effluent	132.5	51.24	2700	kg
CH_4_ emission factor (treatment)	0.14	0.12	0.12	kgCH_4_/kg BOD
N_2_O emission factor (treatment)	0.016	0.0045	0	kgN_2_O-N/kg N
CH_4_ emission factor (discharge)	0.021	0.021	0.021	kgCH_4_/kg BOD
N_2_O emission factor (discharge)	0	0.005	0.005	kgN_2_O-N/kg N
Energy consumed from the grid	86,165,550	3,346,617.6	11,377,219	kWh
Emission factor for grid electricity	0.997	0.097	0.997	kgCO_2_eq/kWh
Sludge removed from wastewater treatment	1,838,687.5	2,372,500	3,677,375	dry weight/kg
BOD_5_ removed as sludge	1,838,687.5	1,898,000	3,677,375	kg
Energy consumed from the grid per month	236,070	278,884.8	948,101.583	kWh/month
Monthly energy costs	4556.51	-	63,206.78	USD/month

**Table 7:** Detailed greenhouse gas assessment data of ECAM in wastewater treatment stage of Strass, Shuozhou and WWTP A of Kunming.

Description	Strass	Shuozhou	WWTP A of Kunming	Unit
Electricity (indirect)	85,907,053	324,622	11,343,087	kg CO_2_eq/year
Treatment process	−8,710,283	−7,735,421	−14,881,290	kg CO_2_eq/year
Discharged water	283.8	198.6	9535	kg CO_2_eq/year
Total GHG wastewater treatment	77,197,054	−7,410,601	−3,528,668	kg CO_2_eq/year

## Data Availability

Not applicable.

## Abbreviations

GHG	Greenhouse gas
ECAM	Energy Performance and Carbon Emission Assessment and Monitoring
WWTP	Wastewater Treatment Plant
CAS	Consumption of Activated Sludge
IPCC	Intergovernmental Panel on Climate Change
IWA	International Water Association
CO_2_	Carbon dioxide
CH_4_	Methane
N_2_O	Nitrous oxide
ICEAS	Intermittent Cycle Extended Aeration
